# Centerband‐Only Detection of Exchange NMR with Natural‐Abundance Correction Reveals an Expanded Unit Cell in Phenylalanine Crystals

**DOI:** 10.1002/cphc.202000517

**Published:** 2020-07-13

**Authors:** Kai Xue, Riza Dervisoglu, Heidrun Sowa, Loren B. Andreas

**Affiliations:** ^1^ Max Planck Institute for Biophysical Chemistry Department of NMR Based Structural Biology Am Fassberg. 11 37077 Goettingen Germany; ^2^ Georg-August-University Goettingen Department of Crystallography Goldschmidtstr. 1 37077 Goettingen Germany

**Keywords:** centerband-only detection of exchange (CODEX), natural-abundance correction, NMR crystallography, phenylalanine crystal, solid-state NMR

## Abstract

The NMR pulse sequence CODEX (centerband‐only detection of exchange) is a widely used method to report on the number of magnetically inequivalent spins that exchange magnetization via spin diffusion. For crystals, this rules out certain symmetries, and the rate of equilibration is sensitive to distances. Here we show that for ^13^C CODEX, consideration of natural abundance spins is necessary for crystals of high complexity, demonstrated here with the amino acid phenylalanine. The NMR data rule out the C_2_ space group that was originally reported for phenylalanine, and are only consistent with a larger unit cell containing eight magnetically inequivalent molecules. Such an expanded cell was recently described based on single crystal data. The large unit cell dictates the use of long spin diffusion times of more than 200 seconds, in order to equilibrate over the entire unit cell volume of 1622 Å^3^.

## Graphical abstract

**NMR crystallography with CODEX**: hundreds of seconds of spin diffusion are used to detect the eight molecules in a large unit cell. Encoded chemical shift anisotropy reveals information hidden in the isotropic spectrum. Accuracy requires correction for natural‐abundance spins in the single‐site carbon‐13‐labelled sample.

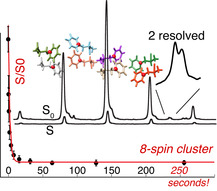

Despite its small size, the common polymorph of phenylalanine that forms upon evaporation from water was reported only in the year 2014, based on high quality single crystals.^1^ The polymorph was previously described as C_2_ with two inequivalent molecules in the unit cell, and now stands corrected with an expanded unit cell containing eight molecules. The corrected structure is also in agreement with a DFT report in which geometry of a unit cell with eight molecules was optimized.^2^ Magic‐angle spinning (MAS) NMR spectra of phenylalanine resolved two inequivalent molecules based on isotropic chemical shits, consistent with C_2_ symmetry, but demonstrated linewidths that are significantly larger than those of the phenylalanine hydrochloride salt (Figure S1). The new interpretation of a P_21_ polymorph explains this additional broadening. Nevertheless, the isotropic chemical shift spectrum cannot resolve the 8 molecules in the unit cell, a degeneracy of chemical shifts well known in NMR crystallography .^3^, ^4^, ^5^

The CODEX pulse sequence is based on magnetization exchange among nearby spins and is more sensitive than multiple quantum^6^, ^7^, ^8^, ^9^ approaches to determine oligomeric number or inequivalent molecules in crystals. The sequence was first invented by Schmidt‐Rohr et al. for investigation of slow dynamics^10^, ^11^ and later demonstrated by Hong et al. for the application of determining oligomeric numbers and intermolecular distances.^12^, ^13^ By isotope labelling only a single site and encoding the orientation dependent chemical shift anisotropy (CSA), the CODEX sequence can detect spin exchange^14^, ^15^ between chemically equivalent but orientationally inequivalent spins. Such exchange is not detected in 2D exchange spectra such as proton assisted spin diffusion (PDSD),^14^, ^15^ since the exchanged signal also occurs on the diagonal. At sufficiently long mixing times, initial magnetization is equally distributed among different orientations in the cluster, and since only the starting orientation results in signal, it follows that the signal plateaus at the inverse of the number of spins in the molecular cluster. In a crystal, translation of the lattice results in an identical orientation, and the signal is retained, allowing the determination of the number of inequivalent molecules in the unit cell.^13^ In addition, if the lattice geometry or oligomer symmetry is known, distances among spins can be deduced from the spin diffusion rate.^13^, ^14^ Due to its robustness in long distance restraint determination, CODEX has been widely used. Hong and her colleagues applied ^13^C and ^19^F CODEX to the determination of spin diffusion rates in single‐site labelled glycine, leucine, phenylalanine hydrochloride and tryptophan crystals^13^, ^16^ and to distance determination in membrane proteins such as the tetrameric Influenza A M2 and HIV gp41 in lipid bilayers.^17^, ^18^, ^19^ Schmidt‐Rohr and Hu quantified the distribution of strongly bonded citrates on the surface of hydroxyapatite using ^13^C CODEX and REDOR.^20^ Kong and coworkers applied ^13^C CODEX on the partitioning of surface ligands on nano‐crystals and established a correlation with solubility of nanoparticles.^21^ In the same year, Kong and Wang applied ^13^C CODEX to measure the distribution of acetate on metal organic framework (MOF) surfaces and describe the potential defects caused by acetate in the MOF structure.^22^

However, previous reports of ^13^C CODEX data considered only spin diffusion taking place between singly labelled carbon sites without consideration of the magnetization exchange to the ^13^C from unlabeled sites, which occurs with a natural abundance of 1 %. Consideration of spins at 1 % abundance is not needed for small molecular size, such that the previous studies are clearly valid. But the effect from unlabeled sites accumulates with the increase in molecular size and with the number of distinct molecular orientations. We take a commonly used amino acid phenylalanine as an example. Apart from the singly labeled site, there are 8 other carbon sites in phenylalanine. The number of spins over which magnetization can equilibrate (M′) therefore averages 1.08 rather than to 1 if we consider unlabeled sites in a single molecule (M=1). Consequently, for a phenylalanine cluster of 10 molecules, the total number of spins to consider is 10.8 in the 10‐molecule cluster, which is very close to 11 spins. The equilibrium magnetization after long mixing time CODEX will therefore result in an incorrect conclusion of an 11‐molecule cluster, unless the natural abundance spins are considered. This impact is much larger than the almost negligible influence from the 99 percent isotope enrichment level (Table S1).

Here we implement a two‐part strategy to accurately account for natural abundance. First, we introduce a premixing period to the original CODEX scheme in order to equilibrate the level of magnetization across all sites, which are initially polarized unequally due to site‐specific cross polarization(CP) efficiency.^23^ Secondly, in the data analysis step, we calculate the average sum of spins considering natural abundance spins at 1 percent. The scheme is applied in analysing phenylalanine crystals labelled at either ^13^C′, or at the ^13^Cγ. The use of very long spin diffusion time up to 512 seconds allows transfer over unprecedented distance between ^13^C spins.

By comparing CODEX curves of three different crystals of increasing complexity (Figure 1), we found that consideration of natural abundance spins becomes important for M=8. In Figure 1A, CODEX curves are shown for glycine and for two polymorphs of phenylalanine. Experimental details and verification of the phenylalanine polymorph is found in the SI text and Figures S2‐S4. The signal (S) and reference signal (S_0_), where *τ_m_* and *τ_z_* values are swapped, can be seen in Figure 1B. The equilibration of initial polarization via long CP and *τ_eq_* (Figure S5) ensures that the reference S_0_ signal accurately compensates for differential T_1_ relaxation or CP efficiency. The T_1_ determination is shown in Figure S6. For glycine(C_2_H_5_NO_2_), each molecule contains one other ^13^C site in natural abundance. Considering two crystal orientations, the average ^13^C cluster spins (M′) is 2.02 (1 % of natural abundance) instead of 2, and the equilibrium that is expected is therefore 0.495 instead of 0.5. Experimentally we determined a value of 0.493+/−0.015 by fitting to a stretched exponential function. The same comparisons are applied for the three crystals in Table 1. For gly and phe•HCl, results are trivial, however, for phe the equilibration is 0.109+/−0.007, and the cluster size appears close to 9 if we do not consider natural abundance. With correction, the experimental and measured equilibrium values agree, within experimental error. Equilibration over the large unit cell indicates relayed transfer over 1622 Å^3^, or a distance of at least 12 Å. The stretched exponential fit accurately determines the equilibrium value, and could even give a distribution of distance restraints.^24^ However, this assumes direct transfer and is not valid for long range spin diffusion, where relayed spin diffusion needs to be considered. This can be done with a rate matrix approach.

**Figure 1:**
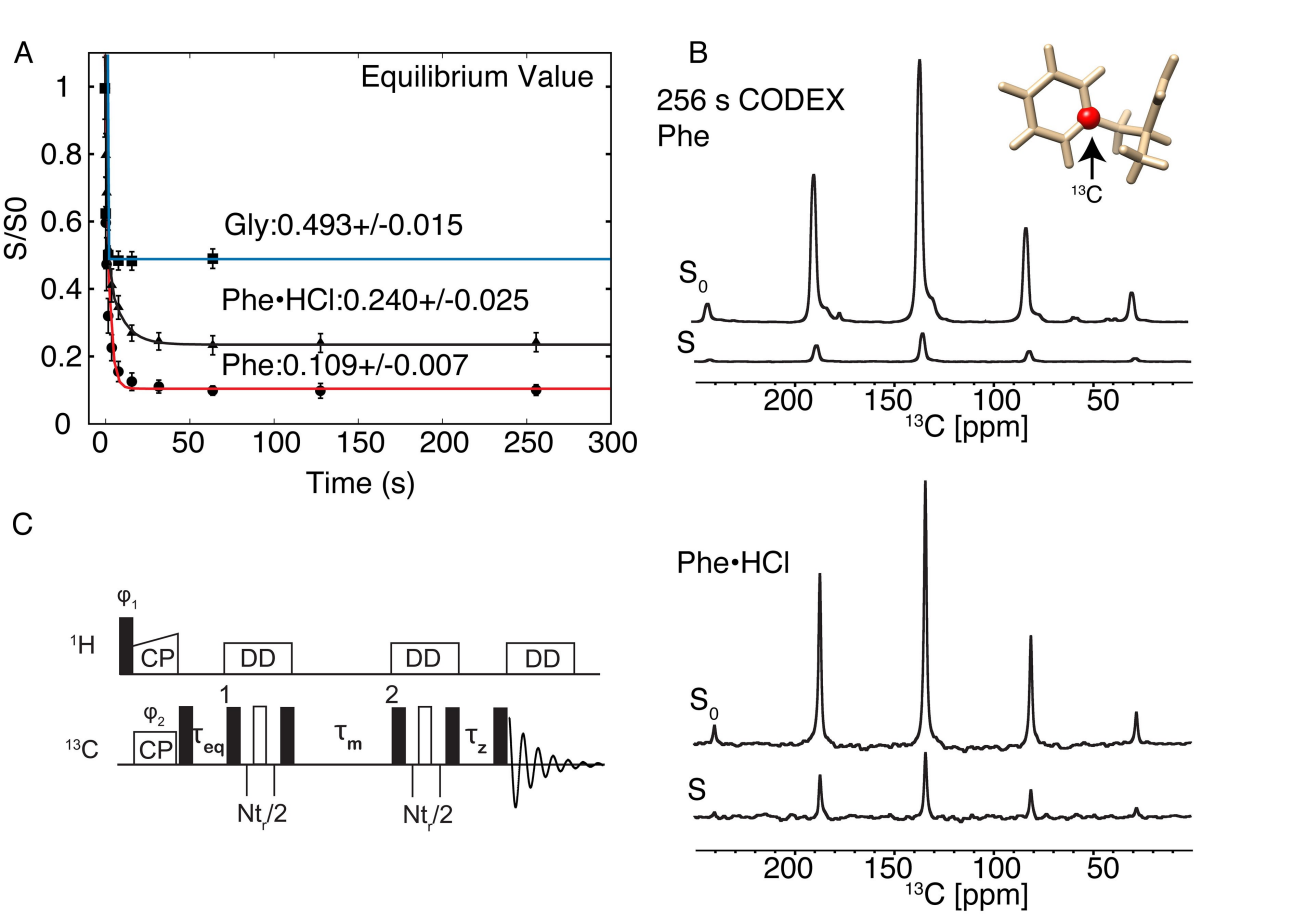
**A)** CODEX decay curves for Glycine (blue), phenylalanine hydrochloride (black) and phenylalanine (red) crystals. Error bars (2σ) were estimated with a Monte Carlo approach and the spectral noise. S is the CODEX signal while S_0_ is the signal to compensate for T_1_ relaxation during CODEX by exchanging τ_eq_ and τ_z_. **B)** Example 1D carbon spectra after 256 s spin diffusion and the labelling scheme for phenylalanine and phenylalanine hydrochloride in **A). C)** A modified version of the CODEX pulse sequence and labelling scheme for phenylalanine and phenylalanine hydrochloride in A). 16 seconds premixing (τ_eq_) was used for phenylalanine, and 5 s for glycine. A long CP of 2.8 ms was used. Spectra were acquired on a 600 MHz spectrometer with 8 kHz MAS at 100 Kelvin.

**Table 1:** Expected and measured equilibrium CODEX values based on the number of inequivalent molecules M and the natural abundance corrected value M′. Glycine was ^13^C labeled at C′ and Phenylalaine at Cγ.

Sample	1/M	1/M′	1/M′_experimental_
Gly	0.5	0.495	0.493±0.015
Phe ⋅ HCl	0.25	0.231	0.24±0.025
Phe	0.125	0.116	0.109±0.007

## Determination of F(0) with the Rate Matrix

Although the spin diffusion rate depends on both orientation and distance, in practice, variation due to orientation has a relatively small impact on extracted distances. Standard practice is to fit rates to known crystal structures, and apply the same calibration of the parameter F(0) (see below) to unknown distance determination. F(0) is the overlap integral describing the probability that single‐quantum transition occurs at the same frequency for two spins, and depends on the details of the CSA, isotropic chemical shift, dipole coupling with proton, the magnetic field, and the spinning frequency.^13^, ^25^ Although F(0) could in principle be calculated, here and in previous implementations,^13^, ^14^, ^16^, ^17^, ^18^, ^19^ it becomes a calibration factor that is set based on a known crystal form. In the case of phenylalanine, crystal forms are known, and the data provides a useful indication of variations in the spin diffusion rate in different crystal orientations, as has been reported for α and γ glycine polymorphs.^26^, ^27^ Using equations 1–3,^13^, ^15^, ^28^ the spin diffusion rate, represented in the form of F(0) can be calculated using the known atom coordinates.

(1) $$M\left(t\right)=e^{-Kt}M\left(0\right)$$

(2) $$k_{ij}=0.5\pi \cdot \omega_{ij}^{2}\cdot F_{ij}\left(0\right)$$

(3) $$k_{ii}=-\sum_{j\neq i}k_{ij}$$

**Figure 2:**
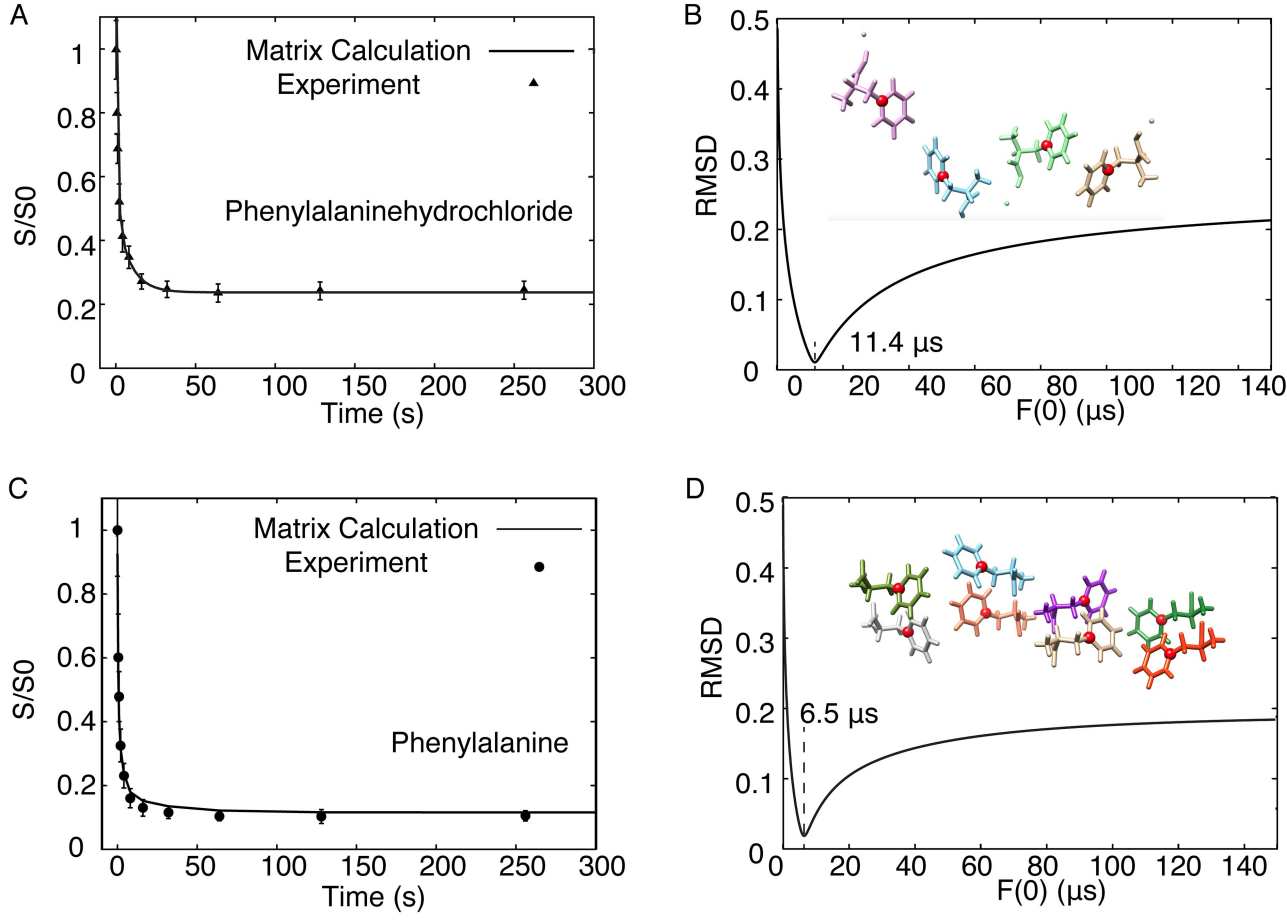
**A**, **C)** ^13^C CODEX decay curves obtained for Phenylalanine hydrochloride and Phenylalanine crystals. **B**, **D)** RMSD of the fit to the data in **A** and **C**, respectively. Respective crystal structures are shown in the inset with the ^13^C labelled sites in red. Spectra were acquired on a 600 MHz spectrometer with 8 kHz MAS at 100 Kelvin.

M stands for the magnetization, K is the rate matrix with elements k_ij_. F_ij_(0) is the overlap integral describing the probability that single‐quantum transition occurs at the same frequency for spin i and j. The rate matrix approach applied to Phe HCl is further described in Figure S7 and the least square fitting of F(0) using the crystal structure coordinates is shown in Figure 2 using home written MATLAB code (see SI).

For ^13^Cγ labelled phenylalanine, we compare F(0) for the two phenylalanine polymorphs in Figure 2. To account for many longer distances present in the crystal, the coupling $\omega_{ij}^{2}$ of Eqn. 2 is replaced by the sum over all spins j within 30 Å of i, where the effective coupling has converged (Figure S8). The closest distance between labelled carbons is 6.09 and 5.00 Å in the hydrochloride^29^ and neutral forms^1^ (CSD entries PHALNC01 and QQQAUJ05), and the dipolar coupling 33.6 and 60 Hz, respectively. We find F(0) values of 11.4 and 6.5 μs.

**Figure 3:**
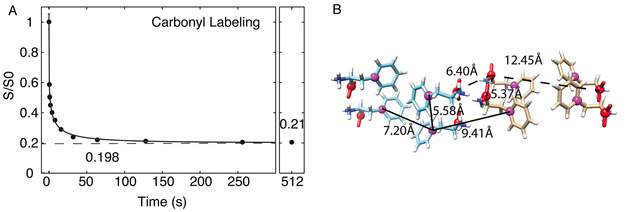
**A)** ^13^C CODEX data of carbonyl ^13^C labelled crystalline phenylalanine fit to a stretched exponential. **B)** Solid lines show Cγ distances, while dashed lines show C′ distances in phenylalanine crystals. Spectra were acquired on a 600 MHz spectrometer with 8 kHz MAS at 100 Kelvin.

A different CODEX equilibrium close to 0.2 is observed for carbonyl labelling (Figure 3) which clearly does not capture the full structure detected at the rings. This can be explained by the packing of phenylalanine molecules in the crystal structure, which results in all the carbonyls lying close to two planes, with a large distance between planes. Four magnetically inequivalent phenylalanine molecules lie in each plane. The closest carbonyl‐carbonyl distance from one plane to the other is 12.5 Å, substantially longer than for the aromatic labelling. Due to the size and complexity of the phe unit cell, this data establishes an upper limit to the maximum spin diffusion distance in ^13^C codex, even for particularly long diffusion times of several minutes, using a moderate MAS frequency at a 600 MHz spectrometer. Indeed, there is a ^13^C‐^13^C separation of 9.4 and 12.5 Å in the ring and carbonyl labelling, respectively, across which spin diffusion must occur to allow full equilibration (Figure 3B). Incomplete equilibration for the carbonyl is also consistent with the range of F(0) values observed (Figure S9, Table S2). Spin pair CODEX curves are shown for the fit carbonyl F(0) value of 1.2 μs in Figure S10.

We showed how careful consideration of natural abundance spins is required to correctly model large spin clusters that occur in the complex unit cell of phenylalanine. The data rule out the original description of phenylalanine crystals with 4 magnetically inequivalent molecules, and are consistent with recent single crystal data that indicate an expanded unit cell with 8 magnetically inequivalent molecules. With carbonyl labelling, we showed that 12.5 Å is an upper limit to carbon spin diffusion at 8 kHz MAS at a 600 MHz spectrometer. This example shows how CODEX data can be useful for refinement of structures if ambiguity exists in diffraction data, and will be useful for analysis of molecule clusters in materials applications.

## Conflict of interest

The authors declare no conflict of interest.

## Supporting information

As a service to our authors and readers, this journal provides supporting information supplied by the authors. Such materials are peer reviewed and may be re‐organized for online delivery, but are not copy‐edited or typeset. Technical support issues arising from supporting information (other than missing files) should be addressed to the authors.

Supplementary

## References

[1] F. S. Ihlefeldt , F. B. Pettersen , A. von Bonin , M. Zawadzka , C. H. Görbitz Angew. Chem. Int. Ed. 2014, 53, 13600–13604; Angew. Chem. **2014**, 126, 13818–13822; 10.1002/anie.201406886 25336255

[2] M. D. King , T. N. Blanton , T. M. Korter Phys. Chem. Chem. Phys. 2012, 14, 1113–1116. 22143120 10.1039/c1cp22831e

[3] R. K. Harris Solid State Sci. 2004, 6, 1025–1037.

[4] R. K. Harris in *Crystallography and NMR: An Overview, Vol*, **2008**.

[5] R. K. Harris , P. Hodgkinson , V. Zorin , J.-N. Dumez , B. Elena-Herrmann , L. Emsley , E. Salager , R. S. Stein Magn. Reson. Chem. 2010, 48, S103-S112. 20589731 10.1002/mrc.2636

[6] Y. S. Yen , A. Pines J. Chem. Phys. 1983, 78, 3579–3582.

[7] J. Baum , A. Pines J. Am. Chem. Soc. 1986, 108, 7447–7454. 22283239 10.1021/ja00284a001

[8] S. Wi , S. J. Hwang Chem. Phys. Lett. 2006, 426, 187–191.

[9] N. Kanwal , H. Colaux , D. M. Dawson , Y. Nishiyama , S. E. Ashbrook Solid State Nucl. Magn. Reson. 2019, 100, 1–10. 30903912 10.1016/j.ssnmr.2019.03.002

[10] K. Schmidt-Rohr, E. R. deAzevedo, T. J. Bonagamba in *Centerband-Only Detection of Exchange (CODEX): Efficient NMR Analysis of Slow Motions in Solids, Vol*, **2007**.

[11] E. R. deAzevedo , W. G. Hu , T. J. Bonagamba , K. Schmidt-Rohr J. Am. Chem. Soc. 1999, 121, 8411–8412.

[12] J. J. Buffy , A. J. Waring , M. Hong J. Am. Chem. Soc. 2005, 127, 4477–4483. 15783230 10.1021/ja043621r

[13] W. Luo , M. Hong J. Am. Chem. Soc. 2006, 128, 7242–7251. 16734478 10.1021/ja0603406

[14] Z. Olender , D. Reichert , A. Müller , H. Zimmermann , R. Poupko , Z. Luz J. Magn. Reson. Ser. A 1996, 120, 31–45.

[15] D. Suter , R. R. Ernst Phys. Rev. B. 1985, 32, 5608–5627. 10.1103/physrevb.32.5608 9937807

[16] M. Roos , T. Wang , A. A. Shcherbakov , M. Hong J. Phys. Chem. B. 2018, 122, 2900–2911. 29486126 10.1021/acs.jpcb.8b00310 PMC6312665

[17] B. Kwon , M. Roos , V. S. Mandala , A. A. Shcherbakov , M. Hong J. Mol. Biol. 2019, 431, 2554–2566. 31082440 10.1016/j.jmb.2019.05.009 PMC6589385

[18] B. Kwon , M. Lee , A. J. Waring , M. Hong J. Am. Chem. Soc. 2018, 140, 8246–8259. 29888593 10.1021/jacs.8b04010 PMC6382510

[19] J. K. Williams , A. A. Shcherbakov , J. Wang , M. Hong J. Biol. Chem. 2017, 292, 17876–17884. 28893910 10.1074/jbc.M117.813998 PMC5663885

[20] Y. Y. Hu , A. Rawal , K. Schmidt-Rohr Proc. Natl. Acad. Sci. USA 2010, 107, 22425–22429. 21127269 10.1073/pnas.1009219107 PMC3012505

[21] Z. Pang , J. Zhang , W. Cao , X. Kong , X. Peng Nat. Commun. 2019, 10. 10.1038/s41467-019-10389-5 PMC6549164 31165734

[22] Y. Fu , Z. Kang , J. Yin , W. Cao , Y. Tu , Q. Wang , X. Kong Nano Lett. 2019, 19, 1618–1624. 30716273 10.1021/acs.nanolett.8b04518

[23] R. L. Johnson , K. Schmidt-Rohr J. Magn. Reson. 2014, 239, 44–49. 24374751 10.1016/j.jmr.2013.11.009

[24] M. N. Berberan-Santos , E. N. Bodunov , B. Valeur Chem. Phys. 2005, 315, 171–182.

[25] D. L. Vanderhart J. Magn. Reson. 1987, 72, 13–47.

[26] R. E. Marsh Acta Crystallogr. 1958, 11, 654–663.

[27] Y. Iitaka Acta Crystallogr. 1961, 14, 1–10.

[28] L. Emsley in *Spin Diffusion for NMR Crystallography, Vol*, **2009**.

[29] A. R. Al-Karaghouli , T. F. Koetzle Acta Crystallogr., Sect. B: Struct. Crystallogr. Cryst. Chem. 1975, 31, 2461–2465.

