# Supplementary material for: Centerband‐Only Detection of Exchange NMR with Natural‐Abundance Correction Reveals an Expanded Unit Cell in Phenylalanine Crystals
Source: Chemphyschem. 2020 Jul 13;21(15):1622–6. doi: 10.1002/cphc.202000517 (PMC7497148; doi:10.1002/cphc.202000517)
Supplement: Supplementary file 1 — Supplementary [file CPHC-21-1622-s001.pdf]

ChemPhysChem

Supporting Information

Centerband-Only Detection of Exchange NMR with Natural-Abundance Correction Reveals an Expanded Unit Cell in Phenylalanine Crystals

Kai Xue, Riza Dervisoglu, Heidrun Sowa, and Loren B. Andreas*

Materials and methods

Carbonyl ^{13}C glycine was purchased from Cambridge Isotope Laboratory (MA USA), as polycrystalline powder and used as received. Single site L-Phenylalanine samples (Cambridge Isotope Laboratory, MA USA) were dissolved in water at ~60 °C. Crystals formed overnight by evaporation from 60 °C. The crystal form matched previous reports type[1-2] as determined using powder x-ray diffraction (PXRD) measurements (See Figure S2 and S3). Phenylalanine hydrochloride crystals were produced by dissolving phenylalanine in minimal 37% hydrochloride, 90 °C, followed by cooling to room temperature. Needle crystals appeared after one day. Dried crystals were crushed and packed in 3.2 mm rotors.

All NMR measurements were acquired on a 600MHz spectrometer with 8kHz MAS at 100 Kelvin. Cross polarization was applied for 2.8 ms, with carbon and proton nutation frequencies of ~62 kHz and ~73kHz, respectively using a 90-100 percent ramp on proton. Three 180° pulses were used in the rotor synchronized pulse train to encode chemical shift anisotropy. The recycle delay was set at 50 s.

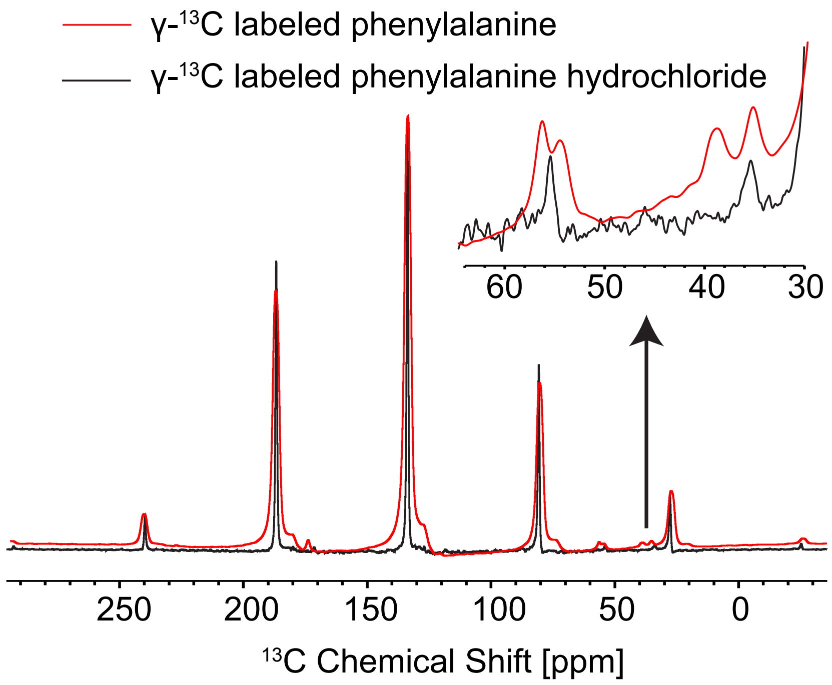

Figure S1: *H-C CP spectrum of phenylalanine crystallites and phenylalanine hydrochloride crystallite. Experiment is carried out with 8kHz MAS at 100 K. Due to polymorphs in crystallite, line width is broader for phenylalanine crystallites and extra splitting is observed at natural abundant aliphatic region.*

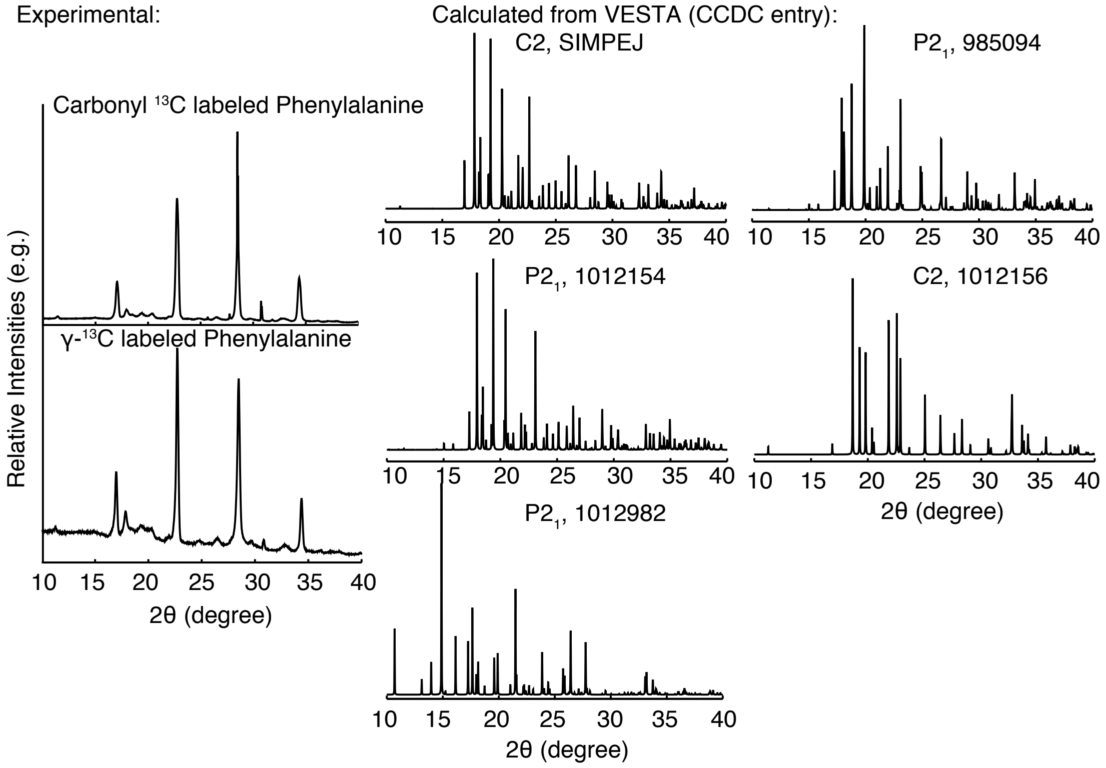

Figure S2: *PXRD data of carbonyl and ring carbon labeled phenylalanine (**Left**). PXRD spectra calculated from structure (**Right**). PXRD data from our sample is different from single molecular computation.*

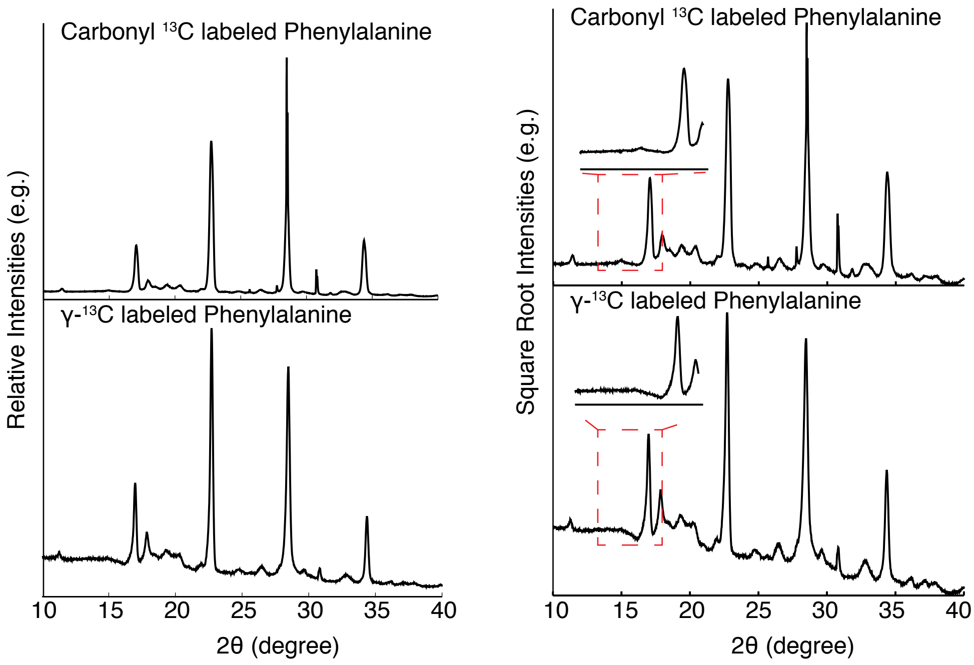

Figure S3: *PXRD data of carbonyl and ring carbon labeled phenylalanine (**Left**). Square root of intensity from original PXRD intensities (**Right**). The weak peak at ~15 degrees is visible in the expansion for a detailed displayed. PXRD pattern shows close similarity with in literature from King et al.* [1]

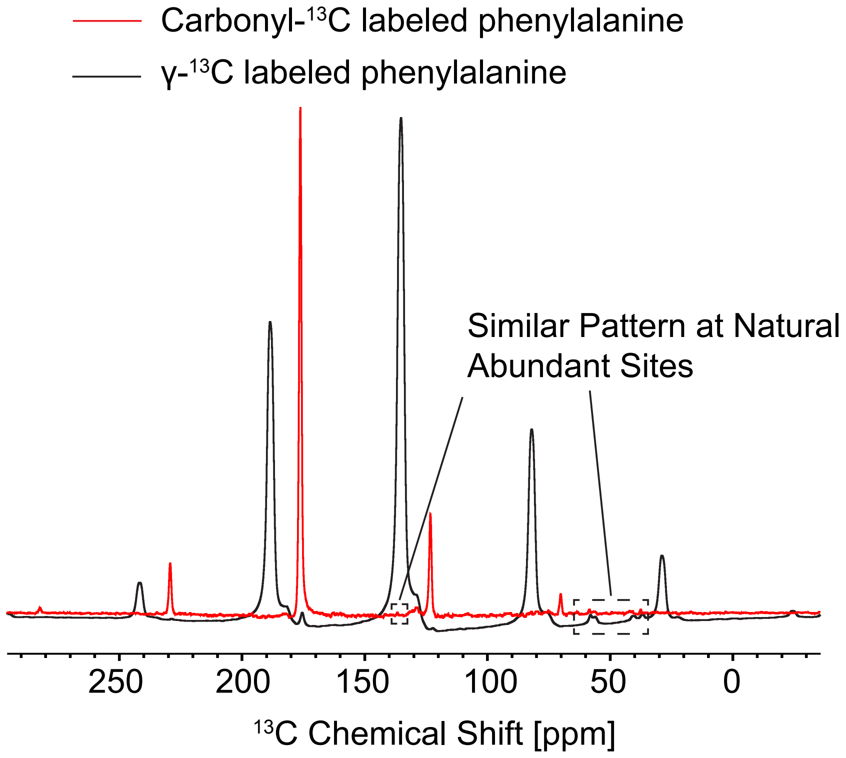

Figure S4: *H-C CP spectrum of phenylalanine crystallites labeled on carbonyl and aromatic ring sites respectively. Experiment is carried out with 8kHz MAS at 100 K. The same chemical shift and peak pattern observed on labeled sites and natural abundant sites indicate the same crystal type in two samples.*

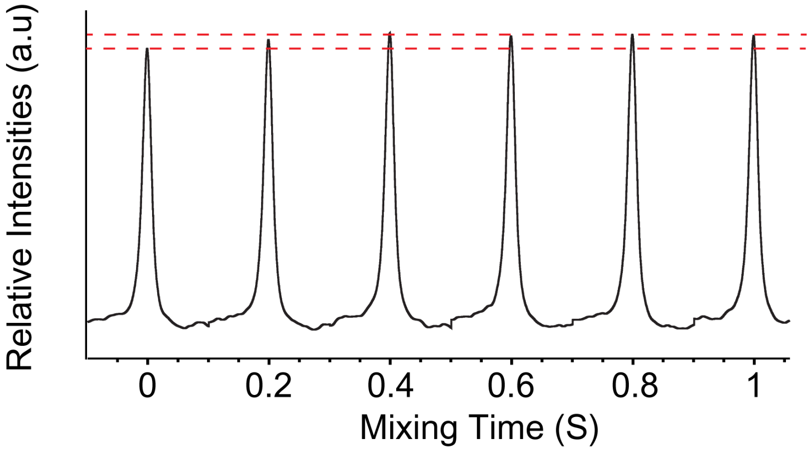

Figure S5: *Equilibrium build up curve for premixing time. Carbonyl labeled phenylalanine was used and pulse sequence was constituted by a CP (300 µs) period and different mixing times. As indicated from the figure, even a very short equilibration time of 0.4 s is enough to reach magnetization equilibrium.*

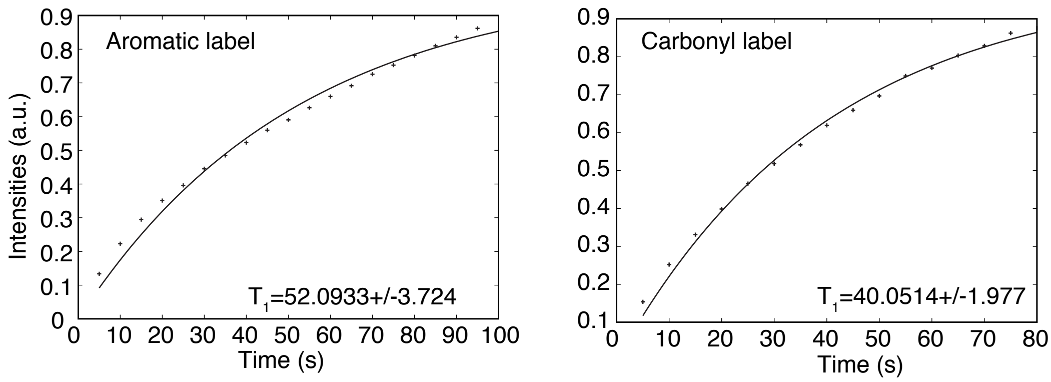

Figure S6: *Carbon T_1 measurement of two phenylalanine samples at 100 K.*

A

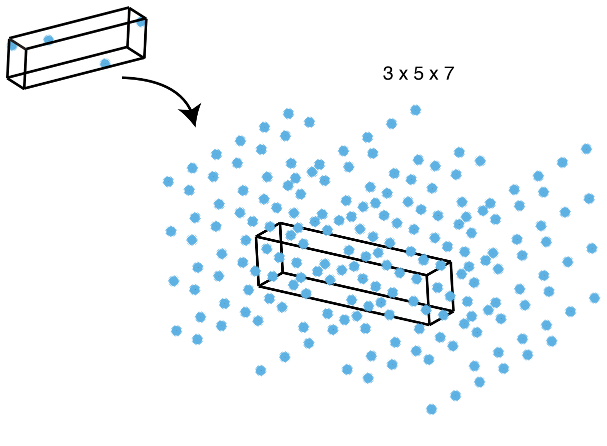

B

$$\mathbf{K}=\begin{pmatrix} K_A & -k_{BA} & -k_{CA} & -k_{DA} \\ -k_{AB} & K_B & -k_{CB} & -k_{DB} \\ -k_{AC} & -k_{BC} & K_C & -k_{DC} \\ -k_{AD} & -k_{BD} & -k_{CD} & K_D \end{pmatrix}$$

$$K_A = k_{AB} + k_{AC} + k_{AD}$$
$$K_B = k_{BA} + k_{BC} + k_{BD}$$
$$K_C = k_{CA} + k_{CB} + k_{CD}$$
$$K_D = k_{DA} + k_{DB} + k_{DC}$$

Figure S7: *Labelled atom coordinates for the crystal structure of Phe HCl is shown in (A) with 105 repeating units. The second moment of dipolar coupling converges by 15 Å. In (B),*

*the 4*4 matrix of rate constants is shown. Matrix elements used to simulate CODEX curves were derived by fitting to a single F(0) value.*

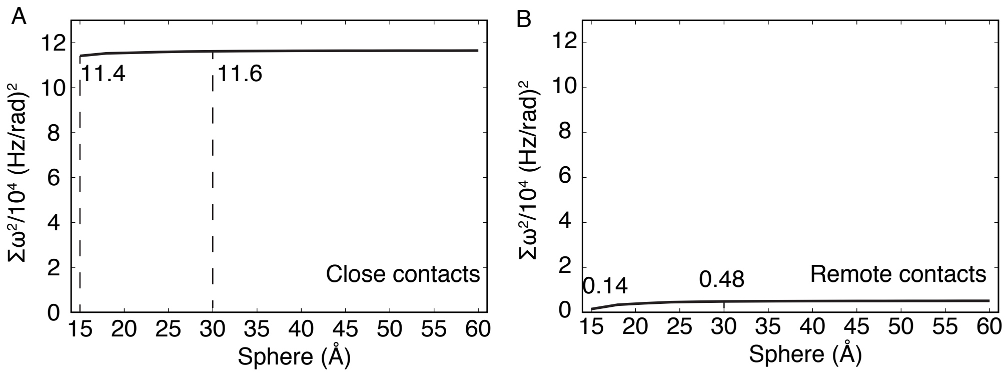

Figure S8: *Sum of the dipolar coupling second moment. A) Sum for two asymmetric sites in the PheHCl crystal with closest separation of ~6 Å, B). Sum for two asymmetric sites in the PheHCl crystal with closest separation of 9.4 Å. To sum up the dipolar coupling second moment, 30 Å is enough in case of PheHCl crystals.*

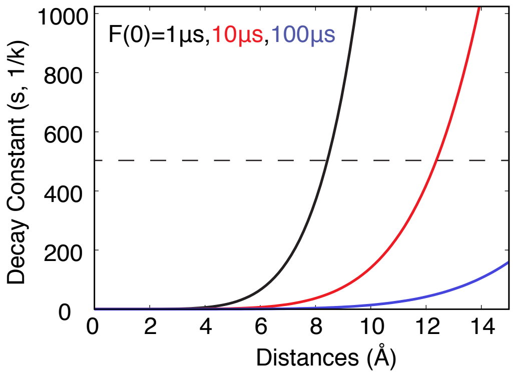

Figure S9: Decay time constant calculated at different F(0) for direct ^{13}C-^{13}C spin transfer.

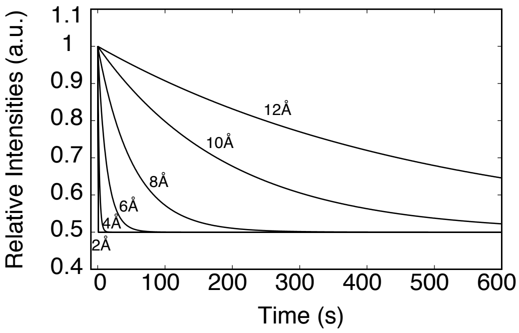

Figure S10: *Simulated CODEX decay curve using a two-spin mode and F(0) of 1.2us, the value determined for carbonyl labeling.*

Number of spins	Expected Equilibrium at 100% enrichment	Expected Equilibrium at 99% enrichment
2	0.5	0.505
4	0.25	0.2525
8	0.125	0.12625

Table S1: Expected equilibrium considering imperfect isotope enrichment on labeled sites. No natural abundance sites are included. Only the first order approximation of 1 site missing is considered since for 99% enrichment, contributions from smaller clusters is negligible.

<table>
<tr><th>Crystal</th><th>Label-Sites</th><th>$\sum\omega_{ij}^2(Hz^2)$</th><th>F(0)</th></tr>
<tr><td>α-glycine</td><td>carbonyl</td><td>1.3*10⁶</td><td>4.6 µs</td></tr>
<tr><td>Phe</td><td>carbonyl</td><td>
<table>
<tr><th></th><th>1</th><th>2</th><th>3</th><th>4</th><th>5</th><th>6</th><th>7</th><th>8</th></tr>
<tr><td>1</td><td>0</td><td>1.9703e+05</td><td>4.7235e+05</td><td>2.4216e+06</td><td>0</td><td>0</td><td>0</td><td>0</td></tr>
<tr><td>2</td><td>1.9703e+05</td><td>0</td><td>2.4171e+06</td><td>4.7155e+05</td><td>0</td><td>0</td><td>0</td><td>0</td></tr>
<tr><td>3</td><td>4.7235e+05</td><td>2.4171e+06</td><td>0</td><td>1.8173e+05</td><td>0</td><td>0</td><td>0</td><td>0</td></tr>
<tr><td>4</td><td>2.4216e+06</td><td>4.7155e+05</td><td>1.8173e+05</td><td>0</td><td>0</td><td>0</td><td>0</td><td>0</td></tr>
<tr><td>5</td><td>0</td><td>0</td><td>0</td><td>0</td><td>0</td><td>1.8686e+05</td><td>4.7384e+05</td><td>2.4131e+06</td></tr>
<tr><td>6</td><td>0</td><td>0</td><td>0</td><td>0</td><td>1.8686e+05</td><td>0</td><td>2.4086e+06</td><td>4.7112e+05</td></tr>
<tr><td>7</td><td>0</td><td>0</td><td>0</td><td>0</td><td>4.7384e+05</td><td>2.4086e+06</td><td>0</td><td>1.9147e+05</td></tr>
<tr><td>8</td><td>0</td><td>0</td><td>0</td><td>0</td><td>2.4131e+06</td><td>4.7112e+05</td><td>1.9147e+05</td><td>0</td></tr>
</table>
</td><td>1.2 µs</td></tr>
<tr><td></td><td>1-aromatic</td><td>
<table>
<tr><th></th><th>1</th><th>2</th><th>3</th><th>4</th><th>5</th><th>6</th><th>7</th><th>8</th></tr>
<tr><td>1</td><td>0</td><td>1.2989e+04</td><td>4.8581e+05</td><td>7.0204e+04</td><td>3.8268e+04</td><td>2.2496e+03</td><td>3.2406e+04</td><td>2.3275e+03</td></tr>
<tr><td>2</td><td>1.2989e+04</td><td>0</td><td>1.2932e+04</td><td>3.0945e+05</td><td>2.2820e+03</td><td>3.8264e+04</td><td>2.3275e+03</td><td>3.2367e+04</td></tr>
<tr><td>3</td><td>4.8581e+05</td><td>1.2932e+04</td><td>0</td><td>4.6369e+04</td><td>3.6720e+04</td><td>2.3344e+03</td><td>3.6399e+04</td><td>2.3327e+03</td></tr>
<tr><td>4</td><td>7.0204e+04</td><td>3.0945e+05</td><td>4.6369e+04</td><td>0</td><td>3.3061e+03</td><td>9.5767e+03</td><td>3.1510e+03</td><td>8.8669e+03</td></tr>
<tr><td>5</td><td>3.8268e+04</td><td>2.2820e+03</td><td>3.6720e+04</td><td>3.3061e+03</td><td>0</td><td>1.1492e+04</td><td>5.0287e+05</td><td>1.2945e+04</td></tr>
<tr><td>6</td><td>2.2496e+03</td><td>3.8264e+04</td><td>2.3344e+03</td><td>9.5767e+03</td><td>1.1492e+04</td><td>0</td><td>1.2951e+04</td><td>4.7744e+05</td></tr>
<tr><td>7</td><td>3.2406e+04</td><td>2.3275e+03</td><td>3.6399e+04</td><td>3.1510e+03</td><td>5.0287e+05</td><td>1.2951e+04</td><td>0</td><td>1.3450e+04</td></tr>
<tr><td>8</td><td>2.3275e+03</td><td>3.2367e+04</td><td>2.3327e+03</td><td>8.8669e+03</td><td>1.2945e+04</td><td>4.7744e+05</td><td>1.3450e+04</td><td>0</td></tr>
</table>
</td><td>6.5 µs</td></tr>
<tr><td>PheHCl</td><td>carbonyl</td><td>
<table>
<tr><th></th><th>1</th><th>2</th><th>3</th><th>4</th></tr>
<tr><td>1</td><td>0</td><td>9.5442e+03</td><td>325400</td><td>5.1122e+03</td></tr>
<tr><td>2</td><td>9.5442e+03</td><td>0</td><td>5.1122e+03</td><td>325400</td></tr>
<tr><td>3</td><td>325400</td><td>5.1122e+03</td><td>0</td><td>9.5442e+03</td></tr>
<tr><td>4</td><td>5.1122e+03</td><td>325400</td><td>9.5442e+03</td><td>0</td></tr>
</table>
</td><td>40.9 µs*</td></tr>
<tr><td></td><td>1-aromatic</td><td>
<table>
<tr><th></th><th>1</th><th>2</th><th>3</th><th>4</th></tr>
<tr><td>1</td><td>0</td><td>116000</td><td>18162</td><td>4.7166e+03</td></tr>
<tr><td>2</td><td>116000</td><td>0</td><td>4.6247e+03</td><td>1.1816e+04</td></tr>
<tr><td>3</td><td>18162</td><td>4.6247e+03</td><td>0</td><td>115940</td></tr>
<tr><td>4</td><td>4.7166e+03</td><td>1.1816e+04</td><td>115940</td><td>0</td></tr>
</table>
</td><td>11.4 µs</td></tr>
</table>

Table S2: Second moment calculate from structure and best-fit F(0) values at 8kHz MAS, 90k, 600 MHz magnetic field. For carbonyl PheHCL, data were obtained for 10kHz, 297k.

CODE for optimizing F(0):

```
% calculate dipolar coupling in rad/s in a sphere of 15A for crystal files
%chrystal file taken from CC
clear
tic
k=zeros(8,8);
distance=zeros(8,8);
A=[ , , ];
A1=[0,0,0];
B=[ , , ];
B1=[0,0,0];
C=[ , , ];
C1=[0,0,0];
D=[ , , ];
D1=[0,0,0];
E=[ , , ];
E1=[0,0,0];
F=[ , , ];
F1=[0,0,0];
G=[ , , ];
G1=[0,0,0];
H=[ , , ];
H1=[0,0,0];
DM=[A;B;C;D;E;F;G;H];
%calculate all distances closest distance 5A, closer than the case in FCl

for i=1:8
    for j=1:8
for o=-2:2
    for m=-3:3
        for n=-1:1
            DM1(j,1)=8.7829*o+DM(j,1)-3.74292*n;
            DM1(j,2)=5.59985*m+DM(j,2);
            DM1(j,3)=30.789456*n+DM(j,3);
            Distance(i,j)=sqrt((DM1(j,1)-DM(i,1))^2+(DM1(j,2)-DM(i,2))^2+(DM1(j,3)-
DM(i,3))^2);
            if Distance(i,j) <= 15 && Distance(i,j)~=0
            omega(i,j)=omega(i,j)+((7598/(Distance(i,j)^3))*2*pi)^2;
            end
        end
    end
end
    end
end

fid1=fopen('F0_RMSD','wt');

for temp=1:3000
     F_0=temp/10;
     for i=1:8
         for j=1:8
             k(i,j)=0.5*pi*omega(i,j)*0.8/4*F_0/1000000;
         end
     end


for i=1:8
    k(i,i)=0;
end
%%%%%%%%%%%%%%%%%%%%%%%%%%%%read data from experiment%%%%%%%%%%%%%%%%%%%%
RMSD=100000000;
x1=dlmread('list_8kHz_phenylalanine','\t','A1..A12');
x=x1; % experimental time
y1=dlmread('list_8kHz_phenylalanine','\t','B1..B12');
y2=dlmread('list_8kHz_phenylalanine','\t','D1..D12');
y3=dlmread('list_8kHz_phenylalanine','\t','F1..F12');
y=y1./y2./y3*1.00;
%%%%%%%%%%%%%%%%%%%%%%%%%%%%read data from experiment%%%%%%%%%%%%%%%%%%%%

k_m=sum(k,2);
k=-1.*k;
for i=1:8
    k(i,i)=k_m(i);
end
K=k; %rate matrix
M0=zeros(8,8);
M0(1,1)=1; %initial state
z=[1 2 3 4 5 6 7 8 9 10 11 12];
```

```
error=0; %initiate startup rmsd
for n=2:12
    temp=-1.*K.*x(n);
    temp1=expm(temp);
    Mt= eig(temp1*M0);
    z(n) = sum(Mt);
    error = (z(n)-y(n))^2+error;
end
error=sqrt(error/11);
fprintf(fid1,'%e,%e\n',F_0,error);
end
fid2=fopen('distances','wt');
%for i=1:7
%    for j=i+1:8
%        fprintf(fid2,'%e\n',distance(i,j));
%    end
%end
fclose(fid1);
t=toc;
```

References

[1] M. D. King, T. N. Blanton, T. M. Korter, *Phys. Chem. Chem. Phys.* **2012**, *14*, 1113-1116.

[2] F. S. Ihlefeldt, F. B. Pettersen, A. von Bonin, M. Zawadzka, C. H. Görbitz, *Angew. Chem. Int. Ed.* **2014**, *53*, 13600-13604.
